# Anticancer activity of calyx of *Diospyros kaki* Thunb. through downregulation of cyclin D1 via inducing proteasomal degradation and transcriptional inhibition in human colorectal cancer cells

**DOI:** 10.1186/s12906-017-1954-2

**Published:** 2017-09-05

**Authors:** Su Bin Park, Gwang Hun Park, Hun Min Song, Ho-Jun Son, Yurry Um, Hyun-Seok Kim, Jin Boo Jeong

**Affiliations:** 1 Department of Medicinal Plant Resources, Andong National University, Andong, 36729 Republic of Korea; 2 Forest Medicinal Resources Research Center, National Institute of Forest Science, Yeongju, 36040 Republic of Korea; 3 Department of Food Science & Biotechnology, Kyonggi University, Suwon, 16227 Republic of Korea; 4 Insititute of Agricultural Science and Technology, Andong National University, Andong, 36729 Republic of Korea

**Keywords:** Anticancer, Calyx of persimmon, Cyclin D1, *Diospyros kaki* Thunb., Human colorectal cancer

## Abstract

**Background:**

Although it has been reported to contain high polyphenols, the pharmacological studies of the calyx of *Diospyros kaki* Thunb (DKC) have not been elucidated in detail. In this study, we elucidated anti-cancer activity and potential molecular mechanism of DKC against human colorectal cancer cells.

**Methods:**

Anti-cell proliferative effect of 70% ethanol extracts from the calyx of *Diospyros kaki* (DKC-E70) was evaluated by MTT assay. The effect of DKC-E70 on the expression of cyclin D1 in the protein and mRNA level was evaluated by Western blot and RT-PCR, respectively.

**Results:**

DKC-E70 suppressed the proliferation of human colorectal cancer cell lines such as HCT116, SW480, LoVo and HT-29. Although DKC-E70 decreased cyclin D1 expression in protein and mRNA level, decreased level of cyclin D1 protein by DKC-E70 occurred at the earlier time than that of cyclin D1 mRNA, which indicates that DKC-E70-mediated downregulation of cyclin D1 protein may be a consequence of the induction of degradation and transcriptional inhibition of cyclin D1. In cyclin D1 degradation, we found that cyclin D1 downregulation by DKC-E70 was attenuated in presence of MG132. In addition, DKC-E70 phosphorylated threonine-286 (T286) of cyclin D1 and T286A abolished cyclin D1 downregulation by DKC-E70. We also observed that DKC-E70-mediated T286 phosphorylation and subsequent cyclin D1 degradation was blocked in presence of the inhibitors of ERK1/2, p38 or GSK3β. In cyclin D1 transcriptional inhibition, DKC-E70 inhibited the expression of β-catenin and TCF4, and β–catenin/TCF-dependent luciferase activity.

**Conclusions:**

Our results suggest that DKC-E70 may downregulate cyclin D1 as one of the potential anti-cancer targets through cyclin D1 degradation by T286 phosphorylation dependent on ERK1/2, p38 or GSK3β, and cyclin D1 transcriptional inhibition through Wnt signaling. From these findings, DKC-E70 has potential to be a candidate for the development of chemoprevention or therapeutic agents for human colorectal cancer.

## Background

Although the detection approaches has been advanced, the incidence of human colorectal cancer with high morbidity and mortality rate remains high [1]. The annual incidence of human colorectal cancer is estimated to be ~1 million, with ~500,000 mortalities [2].

In the United States in 2017, about 95,520 cases of human colorectal cancer are expected to be diagnosed and 52,260 cancer deaths are projected to occur due to human colorectal cancer [3]. Thus, many studies for more effective therapy against human colorectal cancer have been performed. Because long-term treatment using synthetic anti-cancer drugs leads to a lot of side effects, current research in developing a novel anti-cancer agent has been focused to the plant derived chemical compound as a prominent source of new compounds for drug development [4]. Indeed, many plants have been reported to exert anti-cancer activity [5–9].

Plant by-products have the potential value to food and pharmaceutical products through various phytochemicals and pharmacological properties [10]. Thus, plant by-products have been focused for the untapped sources of bioactives [11].

*Diospyros kaki* Thunb (Persimmon) has been reported to contain a variety of beneficial compounds such as condensed tannin, carotenoids, vitamin C and polyphenols [12]. In the plant by-products from *Diospyros kaki* Thunb such as peels, seeds and calyx, calyx of *Diospyros kaki* Thunb (DKC) has been reported to contain high polyphenols and be effective for the treatment of intractable hiccups [13, 14]. DKC as a traditional medicine in Korea has been treated to relieve asthma, chronic bronchitis, and cough symptoms [15, 16]. In the study of DKC for the pharmacological properties, DKC has been reported to possess anti-inflammatory effect through suppression of MAP signaling [17]. In this study, we elucidated anti-cancer activity and potential molecular mechanism of DKC against human colorectal cancer cells. We here reported that 70% ethanol extracts from calyx of *Diospyros kaki* Thunb (DKC-E70) suppressed the proliferation of human colorectal cancer cells and downregulated cyclin D1 level through cyclin D1 degradation by T286 phosphorylation dependent on ERK1/2, p38 or GSK3β, and cyclin D1 transcriptional inhibition through Wnt signaling.

## Methods

### Materials

Cell culture media, Dulbecco’s Modified Eagle medium (DMEM)/F-12 1:1 Modified medium (DMEM/F-12) was purchased from Lonza (Walkersville, MD, USA). PD98059, SB203580, LiCl, MG132 and 3-(4,5-dimethylthizaol-2-yl)-2,5-diphenyl tetrazolium bromide (MTT) were purchased from Sigma Aldrich (St. Louis, MO, USA). Antibodies against cyclin D1, phospho-cyclin D1 (T286), HA-tag, β-catenin, TCF4, p-ERK1/2, total-ERK1/2, p-GSK3β, total-GSK3β, p-p38, total-p38 and β-actin were purchased from Cell Signaling (Bervely, MA, USA). All chemicals were purchased from Fisher Scientific, unless otherwise specified.

### Sample extraction

Calyx of *Diospyros kaki* Thunberg (DKC) was purchased from Humanherb, Korea and formally identified by Jin Suk Koo as the professor of Andong National University, Korea. Twenty gram of DKC was extracted with 300 ml of 70% ethanol with shaking for 48 h. After 48 h, the ethanol-soluble fraction was filtered and concentrated to approximately 90 ml volume using a vacuum evaporator and then freeze-dried. The ethanol extracts (2 g, yield percentage: 10%) from DKC was kept in a refrigerator until use.

### Cell culture and treatment

Human colorectal cancer cell lines such as HCT116, SW480, LoVo and HT-29 were purchased from Korean Cell Line Bank (Seoul, Korea) and grown in DMEM/F-12 supplemented with 10% fatal bovine serum (FBS), 100 U/ml penicillin and 100 μg/ml streptomycin. The cells were maintained at 37 °C under a humidified atmosphere of 5% CO_2_. The ethanol extracts from calyx of *Diospyros kaki* Thunberg (DKC-E70) was dissolved in dimethyl sulfoxide (DMSO) and treated to cells. DMSO was used as a vehicle and the final DMSO concentration did not exceed 0.1% (*v*/v).

### Cell proliferation assay

Cell growth was measured using MTT assay system. Briefly, the cells were plated onto 96-well plate and grown overnight. The cells were treated with DKC-E70 for 24 h. Then, the cells were incubated with 50 μl of MTT solution (1 mg/ml) for an additional 2 h. The resulting crystals were dissolved in DMSO. The formation of formazan was measured by reading absorbance at a wavelength of 570 nm.

### Cell cycle analysis

HCT116 cells were plated in a 6-well plate and grown overnight. The cells were treated with DKC-E70 for 24 h. After then, the cells were dissociated with trypsin, washed in cold PBS and fixed with 70% cold ethanol on ice for 30 min. The suspensions were centrifuged at 1500 rpm for 5 min. The pellets were resuspended in a solution containing 50 μg/ml propidium iodide, 1 mg/ml sodium citrate, 0.3 ml nonidet P-40 and 5 μg/ml RNase A and stayed on ice at least 40 min. Then the pellets were analyzed by a flow cytometer.

### Expression vectors

Wild type HA-tagged cyclin D1 and point mutation of T286A of HA-tagged cyclin D1 were provided from Addgene (Cambridge, MA, USA). Transient transfection of the vectors was performed using the PolyJet DNA transfection reagent (SignaGen Laboratories, Ijamsville, MD, USA) according to the manufacturers’ instruction.

### SDS-PAGE and western blot

After DKC-E70 treatment, cells were washed with 1 × phosphate-buffered saline (PBS), and lysed in radioimmunoprecipitation assay (RIPA) buffer (Boston Bio Products, Ashland, MA, USA) supplemented with protease inhibitor cocktail (Sigma-Aldrich) and phosphatase inhibitor cocktail (Sigma-Aldrich), and centrifuged at 15,000×g for 10 min at 4 °C. Protein concentration was determined by the bicinchoninic acid (BCA) protein assay (Pierce, Rockford, IL, USA). The proteins were separated on SDS-PAGE and transferred to PVDF membrane (Bio-Rad Laboratories, Inc., Hercules, CA, USA). The membranes were blocked for non-specific binding with 5% non-fat dry milk in Tris-buffered saline containing 0.05% Tween 20 (TBS-T) for 1 h at room temperature and then incubated with specific primary antibodies in 5% non-fat dry milk at 4 °C overnight. After three washes with TBS-T, the blots were incubated with horse radish peroxidase (HRP)-conjugated immunoglobulin G (IgG) for 1 h at room temperature and chemiluminescence was detected with ECL Western blotting substrate (Amersham Biosciences, Piscataway, NJ, USA) and visualized in Polaroid film.

### Reverse transcriptase-polymerase chain reaction (RT-PCR)

Total RNA was prepared using a RNeasy Mini Kit (Qiagen, Valencia, CA, USA) and total RNA (1 μg) was reverse-transcribed using a Verso cDNA Kit (Thermo Scientific, Pittsburgh, PA, USA) according to the manufacturer’s protocol for cDNA synthesis. PCR was carried out using PCR Master Mix Kit (Promega, Madison, WI, USA) with human primers for cyclin D1 and GAPDH as followed: cyclin D1: forward 5′-aactacctggaccgcttcct-3′ and reverse 5′-ccacttgagcttgttcacca-3′, GAPDH: forward 5′-acccagaagactgtggatgg-3′ and reverse 5′-ttctagacggcaggtcaggt-3′.

### Transient transfection and luciferase activity

Transient transfection was performed using the PolyJet DNA transfection reagent (SignaGen Laboratories, Ijamsville, MD, USA) according to the manufacturers’ instruction. Cells were plated in 12-well plates at a concentration of 2 × 10^5^ cells/well. After growth overnight, plasmid mixtures containing 1 μg of TOP-FLASH or FOP-FLASH luciferase constructs (Addgene, Cambridge, MA, USA) and 0.1 μg of pRL-null vector were transfected for 24 h. The transfected cells were treated with DKC-E70 for 24 h. The cells were then harvested in 1 × luciferase lysis buffer, and luciferase activity was normalized to the pRL-null luciferase activity using a dual-luciferase assay kit (Promega, Madison, WI, USA).

### Statistical analysis

All the data are shown as mean ± SEM (standard error of mean). Statistical analysis was performed with one-way ANOVA followed by Dunnett’s test. Differences with *P < 0.05 were considered statistically significant.

## Results

### DKC-E70 suppresses the proliferation of human colorectal cancer cells

**Fig. 1:**
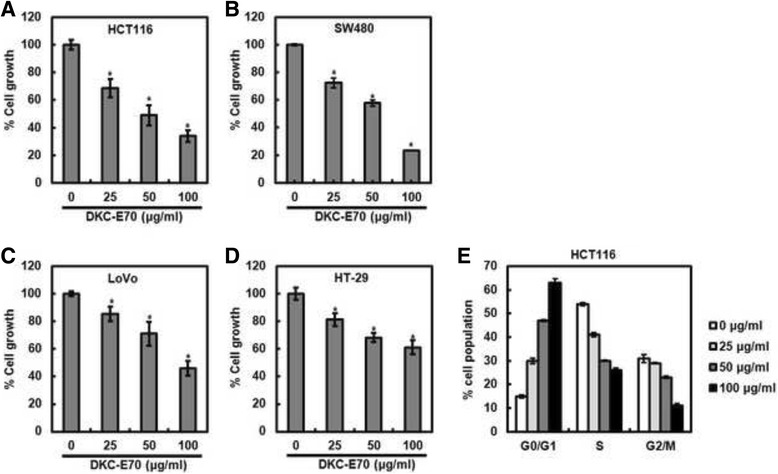
The effect of DKC-E70 on the cell proliferation in human colorectal cancer cells. HCT116 (**a**), SW480 (**b**), LoVo (**c**) or HT-29 cells (**d**) were plated overnight and then treated with DKC-E70 at the indicated concentrations for 24 h. Cell proliferation was measured using MTT assay. *P < 0.05 compared to cell without DKC-E70. **e** HCT116 cells were plated overnight and then treated with DKC-E70 at the indicated concentrations for 24 h. Cell cycle progression was analyzed by flow cytometer

To assess whether DKC-E70 possesses anti-cancer property, we evaluated anti-proliferative activity in human colorectal cancer cell lines such as HCT116, SW480, LoVo and HT-29 cells using MTT assay. As shown in Fig. 1, DKC-E70 suppressed the cell growth by 32% and 28% at 25 μg/ml, 49% and 43% at 50 μg/ml, and 71% and 77% at 100 μg/ml in HCT116 and SW480 cells, respectively (Fig. 1a and b). We also observed that the proliferation of LoVo and HT-29 cells treated with DKC-E70 were reduced by by 15% and 19% at 25 μg/ml, 29% and 32% at 50 μg/ml, and 55% and 39% at 100 μg/ml, respectively (Fig. 1c and d). Next, we investigated whether the inhibition of the cell proliferation by DKC-E70 results from the cell cycle arrest. As shown in Fig. 1e, the majority of HCT116 cells without DKC-E70 were in S phase. However, DKC-E70 treatment dose-dependently induced the accumulation of G0/G1 phase in HCT116 cells.

### DKC-E70 downregulates cyclin D1 expression in both protein and mRNA level in human colorectal cancer cells

**Fig. 2:**
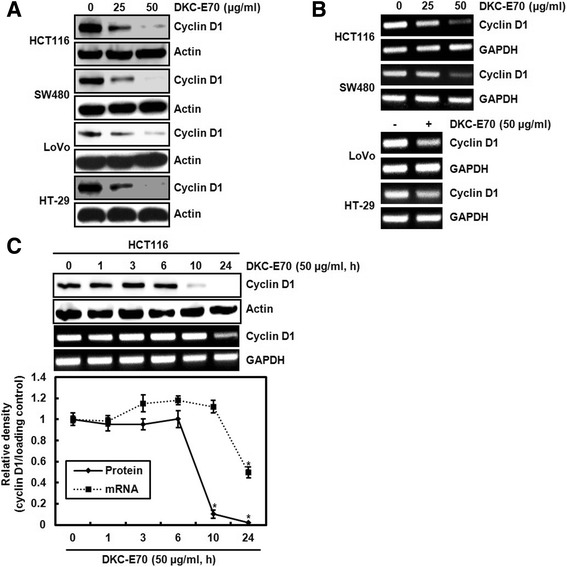
The effect of DKC-E70 on the expression of cyclin D1 at the protein and mRNA level in human colorectal cancer cells. The cells were plated overnight and then treated with DKC-E70 at the indicated concentrations for 24 h. For Western blot analysis (**a**), cell lysates were subjected to SDS-PAGE and the Western blot was performed using antibody against cyclin D1. Actin was used as internal control for Western blot analysis. For RT-PCR analysis of the gene expression of cyclin D1 (**b**), total RNA was prepared. GAPDH was used as internal control for RP-PCR. (**c**) HCT116 cells were treated with DKC-E70 (50 μg/ml) for the indicated times. The protein and mRNA level of cyclin D1 was analyzed using Western blot and RT-PCR, respectively. *P < 0.05 compared to cell without DKC-E70 treatment

Cyclin D1 regarded as one of the proto-oncogenes leads to abnormal cell proliferation through forming the complex with cyclin dependent kinase (CDK) 4/6 [18]. In addition, cyclin D1 overexpression has been reported to be significantly correlated with various cancers including human colorectal cancer [19]. Thus, we investigated the regulatory effect of DKC-E70 on cyclin D1 expression in human colorectal cancer cells. As shown in Fig. 2a, the downregulation of cyclin D1 protein level was observed in the cells treated with DKC-E70. In addition, DKC-E70 reduced the level of cyclin D1 mRNA (Fig. 2b). These data indicate that the transcriptional inhibition may be involved in the downregulation of cyclin D1 protein level mediated by DKC-E70.

In time-course experiment, cyclin D1 protein level was decreased at 10 h after DKC-E70 treatment, while the reduction of cyclin D1 mRNA level by DKC-E70 was observed at 24 h (Fig. 2c). These data suggest that cyclin D1 downregulation by DKC-E70 may be attributed to proteasomal degradation as well as transcriptional inhibition.

### DKC-E70 induces cyclin D1 proteasomal degradation dependent on ERK1/2, p38, JNK and GSK3β

**Fig. 3:**
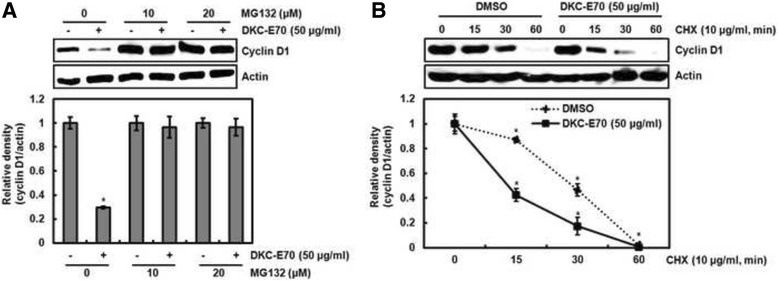
DKC-E70 mediates cyclin D1 degradation. **a** HCT116 cells were pretreated with MG132 at the indicated concentration for 2 h, and then co-treated with DKC-E70 (50 μg/ml) for 10 h. Cell lysates were subjected to SDS-PAGE and the Western blot was performed using antibody against cyclin D1. **b** HCT116 ells were pretreated with DMSO or DKC-E70 (50 μg/ml), and then co-treated with 10 μg/ml of cycloheximide (CHX) for the indicated times. Cell lysates were subjected to SDS-PAGE and the Western blot was performed using antibody against cyclin D1.Actin was used as internal control for Western blot analysis. *P < 0.05 compared to cell without DKC-E70 treatment

To test whether the downregulation of cyclin D1 level by DKC-E70 is mediated via the proteasomal degradation, the cells were pretreated with MG132 as the proteasome inhibitor and then co-treated with DKC-E70. MG132 has been used to investigate ubiquitin-mediated degradation of cyclin D1 [20]. As shown in Fig. 3a, we observed that MG132 attenuates the downregulation of cyclin D1 by DKC-E70. To verify these results for DKC-E70-mediated cyclin D1 degradation, the cells were-pretreated with DMSO or DKC-E70, and then exposed to cycloheximide. As shown in Fig. 3b, DKC-E70 decreased half-life of cyclin D1 protein in HCT116 cells. These data suggest that downregulation of cyclin D1 protein level by DKC-E70 may be involved in the proteasomal degradation.

**Fig. 4:**
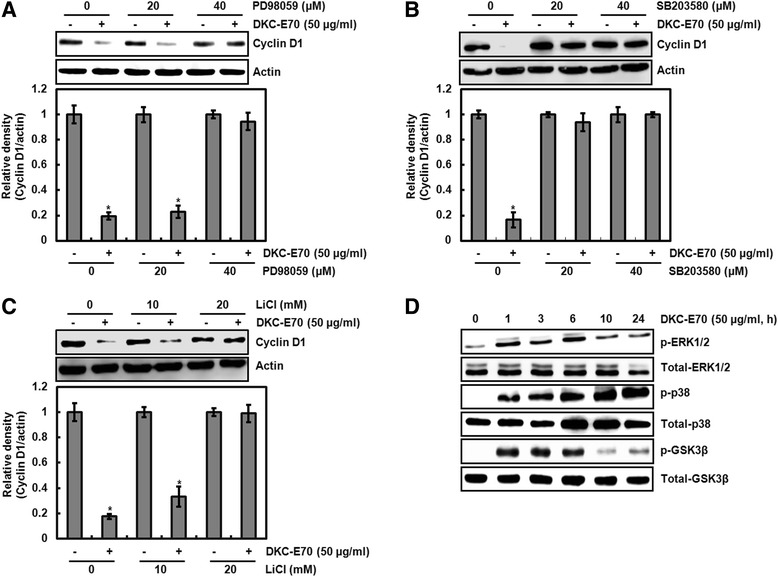
Cyclin D1 degradation by DKC-E70 is dependent on the activation of ERK1/2, p38 and GSK3β. **a**-**c** HCT116 cells were pretreated with PD98059 as an ERK1/2 inhibitor (**a**), SB203580 as a p38 inhibitor (**b**) or LiCl as a GSK3β inhibitor (**c**), and then co-treated with DKC-E70 (50 μg/ml) for 10 h. **d** HCT116 cells were treated with DKC-E70 (50 μg/ml) for the indicated times. Cell lysates were subjected to SDS-PAGE and the Western blot was performed using antibodies against cyclin D1, p-ERK1/2, total-ERK1/2, p-p38, total-p38, p-GSK3β or total-GSK3β. Actin was used as internal control for Western blot analysis. *P < 0.05 compared to cell without DKC-E70 treatment

Cyclin D1 proteasomal degradation has been reported to be associated with the activation of ERK1/2 [21], p38 [22] and GSK3β [23]. Thus, the cells pretreated with PD98059 (ERK1/2 inhibitor), SB203580 (p38 inhibitor) or LiCl (GSK3β inhibitor) and then co-treated with DKC-E70. As shown in Fig. 4a-c, the protein level of cyclin D1 was reduced by DKC-E70 treatment in the cells without the inhibitor treatment such as PD98059, SB203580 and LiCl. However, the reduction of cyclin D1 protein level was blocked in presence of PD98059, SB203580 and LiCl. These data suggest that DKC-E70-mediated cyclin D1 proteasomal degradation may be dependent on the activation of ERK1/2, p38 and GSK3β. To investigate whether DKC-E70 activates ERK1/2, p38 and GSK3β, the phosphorylation of each kinase was analyzed with Western blot. As shown in Fig. 4d, DKC-E70 phosphorylated ERK1/2, p38 and GSK3β.

### DKC-E70-mediated proteasomal degradation of cyclin D1 is preceded by threnonine-286 phosphorylation

**Fig. 5:**
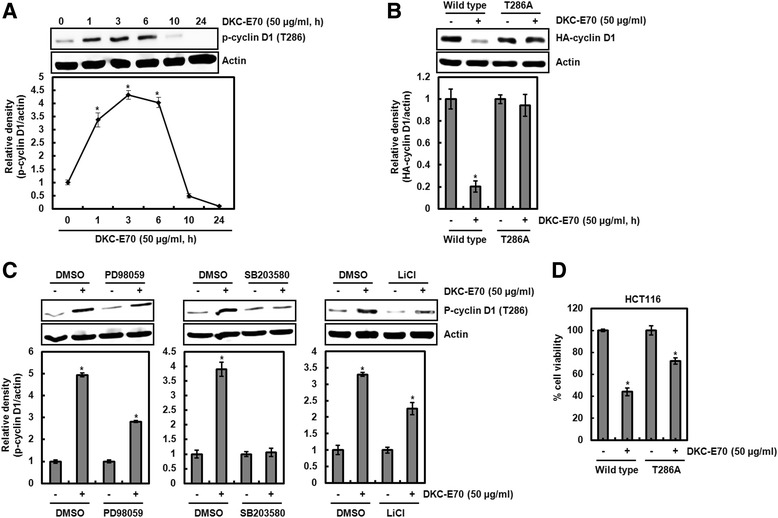
Cyclin D1 degradation by DKC-E70 is followed by T286 phosphorylation dependent on ERK1/2, p38 and GSK3β. **a** HCT116 cells were treated with DKC-E70 (50 μg/ml) for the indicated times. **b** HCT116 cells were transfected with wild type HA-tagged cyclin D1 or HA-tagged T286A cyclin D1 expression vector for 24 h, and then treated with DKC-E70 (50 μg/ml) for 10 h. **c** HCT116 cells were pretreated with PD98059 (40 μM) as an ERK1/2 inhibitor, SB203580 (40 μM) as a p38 inhibitor or LiCl (20 mM) as a GSK3β inhibitor, and then co-treated with DKC-E70 (50 μg/ml) for 3 h. Cell lysates were subjected to SDS-PAGE and the Western blot was performed using antibodies against p-cyclin D1 (T286) or HA-cyclin D1. Actin was used as internal control for Western blot analysis. *P < 0.05 compared to cell without DKC-E70 treatment. **d** HCT116 cells were transfected with wild type HA-tagged cyclin D1 or HA-tagged T286A cyclin D1 expression vector for 24 h, and then treated with DKC-E70 (50 μg/ml) for 24 h. Cell viability was measured using MTT assay. *P < 0.05 compared to cell without DKC-E70

There is a report that threonine-286 (T286) phosphorylation of cyclin D1 increases the rate of cyclin D1 proteasomal degradation [24]. Thus, we firstly tested the effect of DKC-E70 on T286 phosphorylation of cyclin D1. As shown in Fig. 5a, time-dependent increase of cyclin D1 T286 phosphorylation was observed in DKC-E70 treatment. In addition, we investigated whether T286 phosphorylation of cyclin D1 is essential for cyclin D1 proteasomal degradation by DCK-E70 using HA-tagged wild type cyclin D1 and HA-tagged T286A cyclin D1 expression vector. In this assay, the decrease of cyclin D1 by DKC-E70 was not observed in the cells transfected with HA-tagged T286A cyclin D1 compared to the cells transfected with HA-tagged wild type cyclin D1 (Fig. 5b). These data indicate that DKC-E70-mediated cyclin D1 proteasomal degradation may be dependent on T286 phosphorylation.

In Fig. 4, we observed that DKC-E70-mediated cyclin D1 proteasomal degradation may be dependent on the activation of ERK1/2, p38 and GSK3β. Thus, we investigated whether the inhibition of ERK1/2, p38 and GSK3β affects T286 phosphorylation of cyclin D1 by DKC-E70. As shown in Fig. 5c, inhibition of ERK1/2 by PD98059, p38 by SB203580 and GSK3β by LiCl attenuated DKC-E70-mediated T286 phosphorylation of cyclin D1. These data suggest that DKC-E70-mediated proteasomal degradation of cyclin D1 may be preceded by T286 phosphorylation dependent on the activation of ERK1/2, p38 and GSK3β.

In addition, we determined whether DKC-E70-mediated cyclin D1 proteasomal degradation affects the cell viability using HCT116 cells. As shown in Fig. 5d, T286A cyclin D1 transfection partially attenuated the reduction of cell viability induced by DKC-E70 compared to the wild type transfection.

### DKC-E70-mediated transcriptional inhibition of cyclin D1 is attributed to the downregulation of Wnt activation

**Fig. 6:**
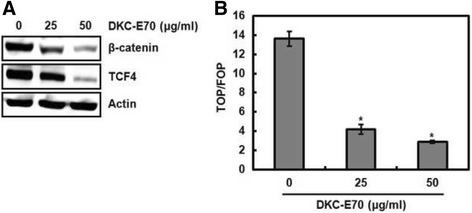
Decreased level of cyclin D1 mRNA by DKC-E70 is attributed to the inhibition of Wnt activation. **a** HCT116 cells were treated with DKC-E70 at the indicated concentrations for 24 h. Cell lysates were subjected to SDS-PAGE and the Western blot was performed using antibodies against β-catenin or TCF4. Actin was used as internal control for Western blot analysis. **b** HCT116 cells were co-transfected with TOP-FLASH or FOP-FLASH constructs containing wild-type or mutated TCF binding sites and pRL-null. The cells were treated with DKC-E70 for 24 h. Luciferase activity for TOP-FLASH and FOP-FLASH was measured as a ratio of firefly luciferase signal/renilla luciferase signal using a dual luciferase assay kit

Wnt signaling pathway has been reported to target cyclin D1 in human colorectal cancer cells [25]. Because we observed that DKC-E70 inhibited the expression of cyclin D1 mRNA, we investigated the effect of DKC-E70 on Wnt signaling using Western blot against β-catenin and TCF4, and β –catenin/TCF-dependent luciferase activity. As shown in Fig. 6a, DKC-E70 dose-dependently inhibited the expression of β-catenin and TCF4. In a luciferase reporter assay using TOP-FLASH or FOP-FLASH constructs containing six copies of wild type or mutated TCF binding sites, TOP/FOP ratio was significantly suppressed in the cells treated with DKC-E70 (Fig. 6b). These findings indicate that DKC-E70-mediated transcriptional inhibition of cyclin D1 may result from the suppression of β-catenin/TCF-dependent signaling.

## Discussion

In cancer development and progression, much attention has been focused on the cyclin D1 as one of the oncogenes associated with the regulation of cell cycle [26]. In cell cycle, cyclin D1 has been reported to induce G1 to S-phase cell cycle transition, which promotes cell proliferation and plays a major role in oncogenesis [27]. Actually, the overexpression of cyclin D1 was observed in many human cancers such as endometrial [28], thyroid [29], urothelial bladder [30], breast [31], brain gliomas [32], esophageal [33] and colorectal cancers [34]. Thus, the regulation of cyclin D1 protein level may be useful for the prevention and treatment of cancer.

Although cyclin D1 overexpression has been regraded to be a common event in the variety of cancer, cyclin D1 overexpression does not occur solely as a consequence of gene amplification. For example, *CCDN1* amplification and cyclin D1 overexpression have been reported to account for 2.5% and 55% in human colorectal cancer, respectively [35]. Indeed, there is growing evidence that the upregulation of cyclin D1 protein level frequently is attributed to its defective regulation at the post-translational level [36, 37]. Because cyclin D1 degradation by many anti-cancer agents has been observed in human cancer cells [38–40], cyclin D1 degradation has been regarded as a useful treatment for anti-cancer. In this study, we suggested two evidences related to the induction of cyclin D1 degradation by DKC-E70. Firstly, decreased level of cyclin D1 protein by DKC-E70 rapidly occurred compared to that of cyclin D1 mRNA. Secondly, MG132 treatment as a proteasome inhibitor abolished DKC-E70-mediated downregulation of cyclin D1 protein level. Lastly, DKC-E70 decreased half-life of cyclin D1 protein in the cells exposed to CHX. These findings indicate that decreased level of cyclin D1 by DKC-E70 may result from its degradation.

Cyclin D1 degradation can be regulated by RxxL motif, and phosphorylation of threonine-286 and -288 [24]. The RxxL motif is associated with anaphase promoting complex-dependent degradation by genotoxic insult [41]. And, cyclin D1 degradation by phosphorylation of threonine-288 has been reported to be mediated by the mirk/Dyrk 1b kinase [42]. In addition, the cyclin D1 stability has been shown to be regulated by threonine-286 (T286) phosphorylation induced by ERK1/2, p38 and GSK3β [21–23, 43, 44]. In this study, we found that DKC-E70 phosphorylated T286 of cyclin D1 and T286A transfection blocked cyclin D1 downregulation by DKC-E70. In addition, it was observed that the inhibition of ERK1/2, p38 and GSK3β associated with T286 phosphorylation attenuated DKC-E70-mediated T286 phosphorylation and subsequent decrease of cyclin D1 protein level. These findings indicate that DKC-E70-mediated cyclin D1 degradation may be attributed to Thr286 phosphorylation dependent on ERK1/2, p38 and GSK3β.

Cyclin D1 overexpression can be regulated by gene amplification through transcriptional activation mediated by Wnt signaling [25]. Interestingly, we observed that DKC-E70 downregulates cyclin D1 mRNA indicating that DKC-E70 may suppress cyclin D1 transcriptional activity. In addition, DKC-E70 downregulated the levels of β-catenin and TCF4, and β –catenin/TCF-dependent luciferase activity. These data indicate that DKC-E70-mediated downregulation of cyclin D1 protein level may be a consequence of the inhibition of gene amplification through suppressing Wnt signaling.

## Conclusions

In conclusion, DKC-E70 downregulates the cyclin D1 level through inducing cyclin D1 degradation by phosphorylation dependent on ERK1/2, p38 and GSK3β and cyclin D1 transcriptional inhibition by blocking Wnt signaling. Complementary alternative medicine has been used for patients with chronic diseases including cancer. Although calyx of *Diospyros kaki* Thunb (DKC) has been treated against asthma, chronic bronchitis and cough symptoms, the anti-cancer activity of DKC has been reported [45]. This study supports the hypothesis that DKC-E70 exerts anticancer property. Our findings will provide the complementary and alternative use of DKC for cancer treatment.

## Abbreviations

CHX: Cyclohexamide
DKC: Calyx of *Diospyros kaki* Thunberg
DKC-E70: 70% ethanol extracts from calyx of *Diospyros kaki* Thunberg
DMSO: Dimethyl sulfoxide
ERK1/2: Extracellular signal–regulated kinase 1/2
GSK3β: Glycogen synthase kinase 3β
MTT: 3-(4,5-dimethylthizaol-2-yl)-2,5-diphenyl tetrazolium bromide
RT-PCR: Reverse transcriptase-polymerase chain reaction
T286: Threonine-286
T286A: Threonine-286 to alanine
TCF4: T-cell factor-4
